# Risk factors for human *Mycobacterium bovis* infections in an urban area of Brazil

**DOI:** 10.1590/0074-02760170445

**Published:** 2018-06-11

**Authors:** Marcio Roberto Silva, Adalgiza da Silva Rocha, Flábio Ribeiro Araújo, Antônio Augusto Fonseca-Júnior, Andrea Padilha de Alencar, Philip Noel Suffys, Ronaldo Rodrigues da Costa, Maria Aparecida Scatamburlo Moreira, Mark Drew Crosland Guimarães

**Affiliations:** 1 Embrapa Gado de Leite, Juiz de Fora, MG, Brasil; 2 Fundação Oswaldo Cruz-Fiocruz, Rio de Janeiro, RJ, Brasil; 3 Embrapa Gado de Corte, Campo Grande, MS, Brasil; 4 Ministério da Agricultura, Pecuária e Abastecimento, Pedro Leopoldo, MG, Brasil; 5 Hospital Regional João Penido, Juiz de Fora, MG, Brasil; 6 Universidade Federal de Viçosa, Viçosa, MG, Brasil; 7 Universidade Federal de Minas Gerais, Belo Horizonte, MG, Brasil

**Keywords:** tuberculosis, zoonoses, risk factors

## Abstract

**BACKGROUND:**

The World Health Organization (WHO) has classified human zoonotic tuberculosis (TB) due to *Mycobacterium bovis* as a neglected issue in the developing world. In a recent cross-sectional study in Brazil, three of 189 TB patients presented with a coinfection of *M. bovis* and *M. tuberculosis* and were selected as cases for this study.

**OBJECTIVE:**

The aim was to evaluate risk factors (RF) for zoonotic TB in an urban area of Brazil in order to guide preventive programmes.

**METHODS:**

A matched case-control study was carried out nested within a cross-sectional study. For each of the three cases, 14 age- and sex-matched controls (TB due to *M. tuberculosis*) were selected.

**FINDINGS:**

Zoonotic potential exposures (ZE) and extrapulmonary TB (EPTB) were independently associated with zoonotic TB in multivariate analyses.

**CONCLUSIONS:**

ZE by occupation and consumption of raw milk and derivative products that place individuals in direct and indirect contact with animals and their excretions/secretions increase the risk for zoonotic TB in Brazil, especially among those with EPTB. Therefore, measures such as efficient control of bovine TB, distribution of pasteurised milk and its derivative products, and the diagnosis and monitoring of zoonotic TB in humans are essential steps, especially in developing countries where bovine TB is enzootic, and further studies are necessary.

In humans, tuberculosis (TB) is a disease caused by species of the *Mycobacterium tuberculosis* complex (MTC), predominantly *M. tuberculosis*, but an unknown proportion of zoonotic TB cases are due to *M. bovis*. This zoonotic pathogen can be transmitted from vertebrates, especially cattle, to humans directly by aerogenous routes or indirectly by the consumption of raw milk or its derivatives (WHO 1994, Thoen et al. 2009).

The incidence rates of this zoonotic disease are generally low on a worldwide level, but available data are a cause for increasing concern about the consequences of this disease in some population groups and settings (Müller et al. 2013). Since HIV-positive individuals are more susceptible to *M. bovis* infections (Thoen et al. 2009), this may potentiate continuous person-to-person transmission cycles of this zoonotic pathogen, as demonstrated among immunosuppressed and even non-immunosuppressed persons (Evans et al. 2007).

There have been outbreaks of multidrug-resistant (MDR) *M. bovis* strains among hospitalised patients with HIV (Samper et al. 1997). These outbreaks highlight the risk associated with MDR *M. bovis*, especially in countries where animals with *M. bovis* and humans with HIV co-exist and are widespread with higher prevalence, particularly in regions such as sub-Saharan Africa, Asia and Latin America (Thoen et al. 2009). In addition, these are regions where the real incidence of *M. bovis* in humans is underestimated or even ignored. The risk of zoonotic TB increases with the practice of consuming non-pasteurised milk and derivative products, which is routine in countries in Africa (Anaelom et al. 2010).

Thus, measures should be developed to identify and control *M. bovis* infection in wild animals and cattle, as these animals may be important natural reservoirs of this pathogen. In addition, animals can be infected by several other *Mycobacterium* species that are pathogenic for humans (Thoen et al. 2009). Therefore, there is a need for medical and veterinary medical professionals to cooperate in outbreaks of these diseases with a “One Health” approach (Wilkins et al. 2008).

Eradication of *M. bovis* in cattle and pasteurisation of dairy products are the cornerstones of prevention of zoonotic TB. Accordingly, control measures for bovine TB based on surveillance in abattoirs, a test-and-slaughter policy, and disease notification have been intensified in the last decades in the countries of South America, notably in Argentina, Brazil, Chile, and Uruguay. In these countries, it is estimated that 2% of the pulmonary TB (PTB) and 8% of the extra-pulmonary cases of TB (EPTB) in humans are caused by *M. bovis* (de Kantor et al. 2014). In Brazil, the proportion of zoonotic TB was 3.5% of 200 cases analysed in 1974 (Corrêa and Corrêa 1974), although more recent studies have shown that it may be lower (Rocha et al. 2011, Silva et al. 2013a).

However, national studies have been restricted mostly to urban regions. Therefore, rural areas should be included (Thoen et al. 2009) in both national and international studies on zoonotic TB to elucidate the dimension of the problem and, when occurring, to identify the main transmission drivers or risk factors in these areas.

Zoonotic TB is still considered a neglected issue in developing areas of the world despite being prioritised by the World Health Assembly WHO as early as the 1950s (Mableson et al. 2014). Furthermore, previous contact with active TB cases (Cleaveland et al. 2007, Bapat et al. 2017), raw milk consumption (Bapat et al. 2017) and HIV infection (LoBue and Moser 2005, Hlavsa et al. 2008) are still found to be important risk factors for this disease. In Brazil, this is also an overlooked issue, but it should not be, since this country is considered an enzootic area for bovine TB, according to the Brazilian Ministry of Agriculture, Livestock and Food Supply. Furthermore, the sale of raw milk is estimated to be approximately 31.7% (Rocha et al. 2014). In addition, sputum acid-fast bacilli (AFB) microscopy and histopathology, which are standard criteria for determining a human TB diagnosis, may potentially overlook zoonotic TB cases.

In a recent cross-sectional study in an urban area of Brazil, we found three (1.6%) of 189 TB patients with coinfection of *M. bovis* and *M. tuberculosis* (Silva et al. 2013a). In the present study, these three cases were compared with a sample of controls from the aforementioned cross-sectional study with the aim of evaluating risk factors for human *M. bovis* infections and in the hope of guiding policy makers regarding preventive programmes and further epidemiological studies on zoonotic TB.

## SUBJECTS AND METHODS

*Study population, region and period* - The study population (TB patients) was recruited between March 2008 and February 2010 from two public referral centres for human TB treatment in Juiz de Fora, Minas Gerais State, Brazil, which has a predominantly urban population (98%) with approximately 500,000 inhabitants. The study area was chosen because it is a macro-regional referral city for TB, including satellite municipalities and rural areas. In addition, it is a city that has an adequate laboratory infrastructure for the cultivation of mycobacteria, with the necessary biosafety standards. Smaller cities and rural locations in Brazil do not generally cultivate mycobacteria for TB diagnosis, which is usually based on sputum smear microscopy only.

In addition to conventional Löweinstein-Jensen (LJ) medium for *M. tuberculosis* growth, Stonebrink with pyruvate-containing egg medium (SB) was included in the routine of this major public referral laboratory in order to facilitate the possible growth of *M. bovis*.

*Epidemiological data collection* - Patients participated in a person-to-person interview, in which a structured questionnaire was used to collect information on a range of variables related to the following: (i) individual and household characteristics (e.g., sex, age, income and housing); (ii) household practices; (iii) types of occupation; and (iv) consumption of milk and/or milk products. Milk and dairy product intake was assessed according to preparation before the consumption of milk (e.g., boiled, soured or raw) and milk products (e.g., cheese made with pasteurised or raw milk). Bacillus Calmette-Guérin (BCG) vaccine status was also recorded.

*Study design and definition and selection of cases and controls* - This matched case-control study was carried out nested within an earlier completed cross-sectional study (Silva et al. 2013a). For each of the three cases that were co-infected with *M. bovis* and *M. tuberculosis*, we selected 14 age- (± 10 years) and sex-matched controls (TB due to *M. tuberculosis*) among the 189 available TB patients characterised in the aforementioned cross-sectional study. In that study, AFB-positive clinical specimens of all patients diagnosed as PTB were inoculated as described above, followed by phenotypical and genotypical analyses for *Mycobacterium* speciation. In the case of patients diagnosed as EPTB, physicians requested cultivation on a subset of the patients only. Additionally, genotypical analyses in formalin-fixed and paraffin-wax-embedded biopsy tissue samples from EPTB patients were attempted to identify MTC carriers (Silva et al. 2013a).

The matching method was used to avoid confounding because it is the strategy of choice in situations with a limited sample size, as in this study. Age and sex variables were used as matching criteria according to a previous study (Cleaveland et al. 2007). However, different from the usual four or five controls per case as is standard practise, in this study, we used the maximum available number of controls. We did this because there are settings in which a higher control-to-case ratio may be desirable, such as when the cost of including additional controls is negligible, there is a limited number of cases available or there is concern for sufficient numbers in stratified analyses (Hennessy et al. 1999); all were applicable in the present study.

*Study variables* - The current cases were those with the presence of *M. bovis*, while controls had *M. tuberculosis* only. The main explanatory variables were lifetime consumption above the median level of raw milk and/or cheese produced from raw milk (i.e., raw-milk cheese) and lifetime zoonotic potential exposures (ZE) to *M. bovis* sources. The lifetime consumption (days) of both raw milk and raw-milk cheese was defined using both the frequency and the period of consumption of these types of diets of bovine origin, reaching a numerical estimate “lifetime consumption” (days). These estimates were based on the reported number of days in one episode and the frequencies of these episodes combined. We classified patients as having ZE if there was (i) direct contact with bovine livestock via aerogenic routes (occupations related to both bovine livestock and the agro-food industry of bovine origin), (ii) consumption above median levels of bovine raw-milk and (iii) consumption above median levels of raw-milk cheeses.

*Statistical analyses (case-control study)* - Data were analysed as a matched case-control study. Conditional logistic regression (CLR) was used to investigate the relationship between an outcome of being a case or a control and a set of prognostic factors.

The case-control analyses were carried out in a two-stage process. First, univariate CLR analyses of all variables of interest were evaluated. Second, variables that presented p-values < 0.10 in the initial screening were evaluated using multivariate CLR. These variables were included in the multivariate CLR following the forward stepwise (likelihood ratio) method. CLR models were performed using SPSS software, version 20.0 (IBM 2011).

In univariate logistic regression analyses, if some types of exposure had all cases exposed, that would generate an indeterminacy in the release of odds ratio (OR) results. To achieve the same goal, we applied a general approach to matched data as stratified methods for sparse data (Greenland 2008). In this approach, each set of matched cases and controls were considered as a stratum. Using this strategy, it was possible to apply Haldane's correction and release approximate maximum-likelihood estimates of the common OR values at the univariate analyses, using OpenEpi software (Dean et al. 2013). Haldane's correction involves adding 0.5 to all of the cells of a contingency table if any of the cell expectations would cause a division by zero error. Accordingly, OR and 95% confidence intervals (CIs) were shown.

In the case of multivariate CLR analyses, if some types of exposure had all cases exposed, that would generate a frequency of zero in any single cell of the table, which implies quasi-complete separation. When quasi-complete separation happens, it prevents the convergence of the maximum likelihood estimates for the coefficients. In that case, we only reported likelihood ratio chi-squares for complete and partial models. In all CLR analyses, p ≤ 0.05 was taken to indicate significance. The software Epi Info version 3.5.3 (Dean et al. 1994) was used for data management.

*Ethics* - The study was approved by the Research Ethics Committee of the Federal University of Juiz de Fora (protocol 819.125.2006) and João Penido Regional Hospital (protocol 52/08). Participants were informed about the objectives of the study, and written informed consent was obtained.

Patients with a positive diagnosis of TB and HIV received therapy available from the government medical services. All patients were provided with pre-test counselling prior to being asked for permission to carry out HIV testing and post-test counselling, according to guidelines on ethics for health research.

## RESULTS

*Description of the study population* - The prevalence (1.6%) of *M. bovis* among a sample of 189 individuals diagnosed with TB in Juiz de Fora, an urban area of Brazil, and descriptive characteristics of that population have previously been reported (Silva et al. 2013a). That sample was the basis for the current nested matched case-control study. Briefly, 186 (98.4%) out of 189 patients presented with only *M. tuberculosis*, and three (1.6%) also presented with *M. bovis* in addition to *M. tuberculosis.* In the sample, 185 patients (97.4%) lived in an urban area of Juiz de Fora. Altogether, 15 patients (7.9%), 72 (38.1%) and 46 (24.3%) were current, former and not consumers of bovine raw milk, respectively, and 56 (29.7%) did not have such information recorded. Regarding derivative dairy products, 59 (31.2%), 51 (27%) and 23 (12.1%) were current, former and not consumers of bovine raw-milk cheeses, and 56 (29.7%) did not have such information recorded. As for bovine contact, 65 patients (34.4%) had a history of occupations related to bovine livestock or agro-food industries of bovine origin while 67 (35.5%) did not; 57 (30.1%) did not have such information recorded. Regarding HIV status, 86 (45.5%) patients were HIV-negative and 24 (12.7%) were HIV-positive; 79 (41.8%) did not consent to any serological diagnosis for HIV. The minimum, median and maximum lifetime consumption values were zero, 15 and 20,832 days for bovine raw milk and zero, 279 and 21,840 days for bovine raw-milk cheese, respectively. Table I shows the proportions of exposures to each possible variable by co-infected *M. bovis-M. tuberculosis* patients (n = 3) and *M. tuberculosis* patients (n = 42) selected for the matched case-control study.

**TABLE I:** Univariate conditional logistic analyses for *Mycobacterium bovis* infections in Brazil

Variables	Exposures		CMLE OR (CI 95%)	Score (p value)	Likelihood ratio (p value)	Adjusted Mid-P exact (one tail)
	Cases	Controls				
Consumption above median levels of raw-milk cheese (Yes/No)	3/3	19/42	3.58 (2.02-24.13)*	3.67 (0.055)	4.70 (0.030)	0.048
Zoonotic potential exposures (Yes/No)	3/3	14/42	5.71 (2.827-40.99)*	5.09 (0.024)	5.92 (0.015)	0.024
Clinical features of tuberculosis (TB) (extrapulmonary/pulmonary)	2/3	5/42	13.57 (1.10-167.28)	6.29 (0.012)	4.30 (0.038)	0.029
History of housing in rural areas (Yes/No)	0/3	14/42	–	1.40 (0.230)	2.20 (0.130)	–
Consumption above median levels of raw milk (Yes/No)	0/3	19/40	–	2.46 (0.110)	3.49 (0.062)	–
HIV/AIDS (Yes/No)	0/3	4/34	–	0.13 (0.71)	0.12 (0.72)	–
BCG Vaccine (Yes/No)	1/3	9/42	–	0.15 (0.69)	0.14 (0.70)	–

BCG: Bacillus Calmette-Guérin (BCG); CMLE OR: conditional maximum-likelihood estimate of the odds ratio;

* approximate OR (with Haldane's correction).

*Risk factors for M. bovis infections* - The univariate CLR analyses identified that individuals who had EPTB (p = 0.029) had lifetime consumption above median levels of bovine raw milk cheeses (p = 0.048) and reported ZE (p = 0.024) were significantly more likely to be *M. bovis*-positive cases (Table I).

After adjustment for potential confounding factors, two variables remained independently (p = 0.005) associated with zoonotic TB: EPTB and potential zoonotic exposures. This final multivariate model containing two variables (EPTB and potential zoonotic exposures) showed significant changes (p = 0.012) compared to the previous step (only the EPTB variable included). Table II shows that only likelihood ratio chi-squares for the complete and partial models were presented instead of maximum likelihood estimates for the coefficients, because a quasi-complete separation condition was detected.

**TABLE II:** Multivariate conditional logistic analyses for *Mycobacterium bovis* infections in Brazil

Block	Step (variable entered)	-2 Log Likelihood	Overall (score) Chi-square (p-value)	Change from previous step Chi-square (p-value)	Change from previous block Chi-square (p-value)
0*	0 (variables not in the equation)	16.248	–	–	–
1**	1*** [extrapulmoary clinical features of tuberculosis (TB)]	11.944	6.298 (0.012)	4.304 (0.038)	4.304 (0.038)
1**	2**** (zoonotic potential exposures and extrapulmonary clinical features of TB)	5.576	8.848 (0.012)	6.368 (0.012)	10.673 (0.005)

* beginning block number 0, initial Log Likelihood function: −2 Log likelihood: 16.248;

** beginning block number 1, forward stepwise (likelihood ratio) method;

*** variable entered at step number 1: extrapulmonary clinical features of TB;

**** variables entered at step number 2: zoonotic potential exposures.

## DISCUSSION

This is the first study to evaluate risk factors for zoonotic TB in Brazil. Despite the small number of cases, risk factors associated with zoonotic TB could be identified and included EPTB and ZE by occupation and the consumption of raw milk and derivative products that place individuals in direct and indirect contact with animals and their excretions/secretions. An African study, also presenting sampling size limitation, was likewise successful in finding risk factors using a similar epidemiological design (Cleaveland et al. 2007).

In corroboration with our results, a study conducted in a hospital in Buenos Aires, Argentina, also found high levels (93%) of typical zoonotic exposures for *M. bovis* among patients diagnosed with zoonotic TB, highlighting occupational exposure (65%), a history of living in rural areas (31%) and consumption of raw milk (4%) (Cordova et al. 2012).

Consumption of raw milk and derivative products have also been found to be important risk factors for zoonotic TB by several other studies (LoBue and Moser 2005, Hlavsa et al. 2008, Bapat et al. 2017). Furthermore, the spread of *M. bovis* from animals to humans in developing countries remains a reality, mostly from infected milk (Anaelom et al. 2010).

The prevalence of zoonotic TB has increased along the United States (US) - Mexico border and is linked to consumption of raw-milk cheese (Hlavsa et al. 2008). The link in Brazil was reinforced in this study because this type of food and ZE were associated with *M. bovis*-positive cases by univariate and multivariate analyses, respectively. The detection of *M. bovis* in Brazilian raw-milk cheese by our team and others (Silva et al. 2013b, Cezar et al. 2016) reinforces this epidemiological evidence. In Brazil, raw-milk cheese is very common, and, currently, it is being released for consumption by specific laws (MAPA 2013). However, there is concern that good agricultural practices and good manufacturing practices for raw-milk cheese are not yet widespread in this country.

Several studies in the US have shown an independent association of TB caused by *M. bovis* with both EPTB and HIV coinfection (LoBue and Moser 2005, Hlavsa et al. 2008). The EPTB association, also found in this study, suggests that new cases of zoonotic TB continue to occur in the Americas, rather than being instances of reactivation. Currently, in other industrialised countries, most of the zoonotic TB cases show PTB and are mostly a reactivation of long-standing latent lesions. However, no significant association between *M. bovis* infection and HIV status was identified in this study (all cases were HIV negative) or in surveys of Africa, Canada and Latin America (Müller et al. 2013).

The real incidence of *M. bovis* in humans continues to be roughly underestimated or even ignored in Brazil and other developing countries due to the scarcity of appropriate laboratory facilities to isolate and differentiate *M. bovis* (Thoen et al. 2009). This also hampers the construction of more robust models of the risk factors for zoonotic tuberculosis, as verified also in the present study. We could not include a number of patients in our baseline study because only the higher macro-regional TB referral laboratory that received most of patients prepared pyruvate-containing media (Silva et al. 2013a). It would be an important achievement of the Brazilian Health Ministry to increase the use of adequate culture medium for the detection of *M. bovis*; this was done in Argentina, and it improved the accuracy of information on zoonotic TB there (Barrera and Kantor 1987). This would not only benefit the identification of *M. bovis* but also identification of drug resistant strains, thus, having a wider impact on TB control (Samper et al. 1997).

Our previous observation about coinfection of *M. bovis* and *M. tuberculosis* (Silva et al. 2013b) has also been reported by other studies (Ordoñez et al. 1999, Shah et al. 2006). The diagnostic criteria for the definition of these three zoonotic cases had some limitations that were stated in the previous cross-sectional study (Silva et al. 2013a). We will be briefly point them out, as follows. The confirmation of *M. bovis* infections in two patients with suggestive lesions of EPTB were based only on amplification of specific DNA extracted from formalin-fixed and paraffin wax-embedded biopsy tissue samples and, further, its sequencing. Additionally, the confirmation of *M. bovis* infections in the patient with PTB was based on colony morphology on solid media (flat, smooth and non-pigmented), scantily grown only in SB with pyruvate and phenotypical analyses (especially pyrazinamide resistance). However, this isolate did not show an expected specific gene amplification, as also occurred in the first attempts for 13% of the isolates that had *M. tuberculosis* molecular profiles in that cross-sectional study; these had to be redone. However, the sample diagnosed as *M. bovis*, inactivated by heat, was of insufficient quantity for a new DNA extraction.

Clinical and epidemiological evidence strengthens the zoonotic cases found. Individuals who had ZE were significantly more likely to have zoonotic cases: two out of three zoonotic TB cases in this study occurred in patients who were current consumers of raw milk cheese and presented EPTB; further, one patient had worked with goats and at an abattoir and presented PTB. An EPTB patient took the medication correctly, had been cured, but developed recurrent TB one year after the end of treatment; the molecular result of *M. bovis* was used by the physician to redefine the treatment. Moreover, the PTB patient developed chronic TB and died. Accordingly, *M. bovis* is intrinsically resistant to pyrazinamide and, therefore, may be more involved in TB relapses or chronic infections (Ordoñez et al. 1999).

The most efficient way to control zoonotic TB is through the control/eradication of bovine TB at the herd level. Accordingly, in 2001, Brazil launched the National Program for Control and Eradication of Animal Brucellosis and Tuberculosis. After 15 years, the progress of this programme has been limited by the difficulty in engaging the beef and dairy production chains as true partners in the process (Ferreira Neto et al. 2016).

In conclusion, zoonotic exposures increase the risk for zoonotic TB due to *M. bovis* in Brazil, especially among EPTB patients. Therefore, measures such as efficient control of bovine TB, dissemination of pasteurised of milk and its derivative products, and the diagnosis and monitoring of zoonotic TB in humans are essential. These steps are especially important in developing countries such as Brazil, where bovine TB is enzootic, and further studies are necessary to more accurately estimate prevalence and evaluate risk factors for zoonotic TB with more robust multivariate logistic models.
